# Supplementary figures and images for: Superhydrophilic graphene oxide/electrospun cellulose nanofibre for efficient adsorption of organophosphorus pesticides from environmental samples
Source: R Soc Open Sci. 2020 Mar 11;7(3):192050. doi: 10.1098/rsos.192050 (PMC7137939; doi:10.1098/rsos.192050)

## Slide 1
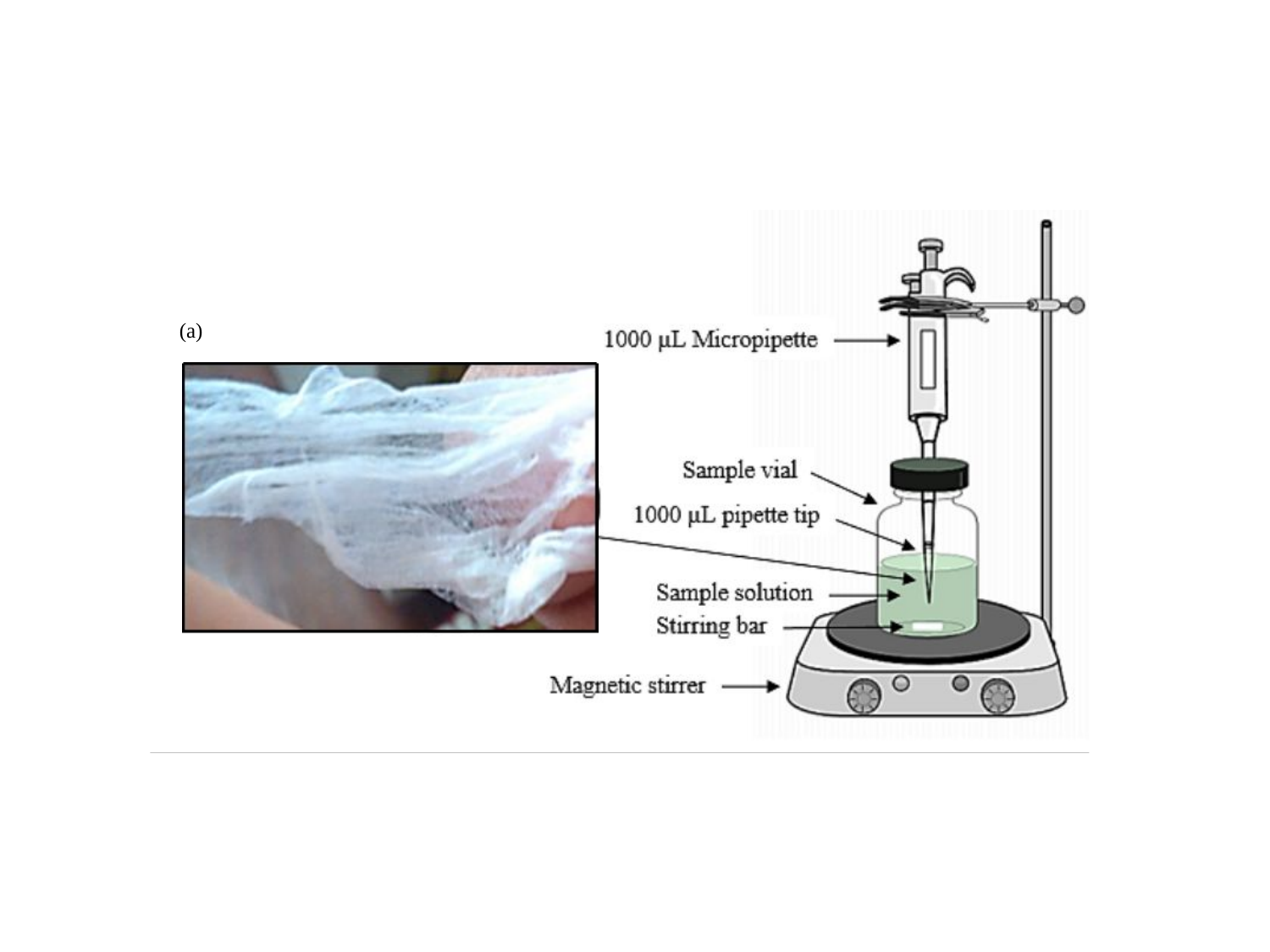

(a)

## Slide 2
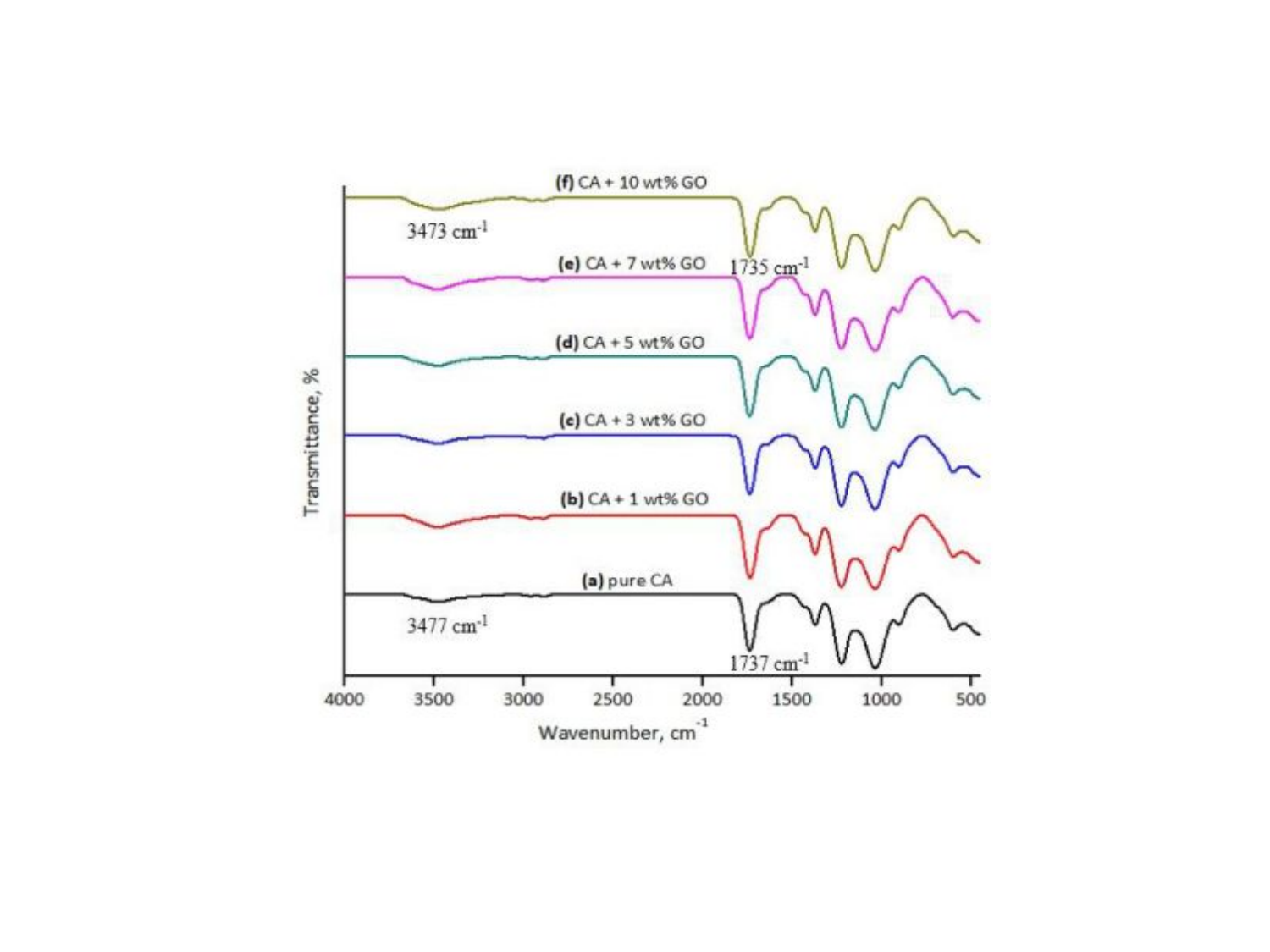

## Slide 3
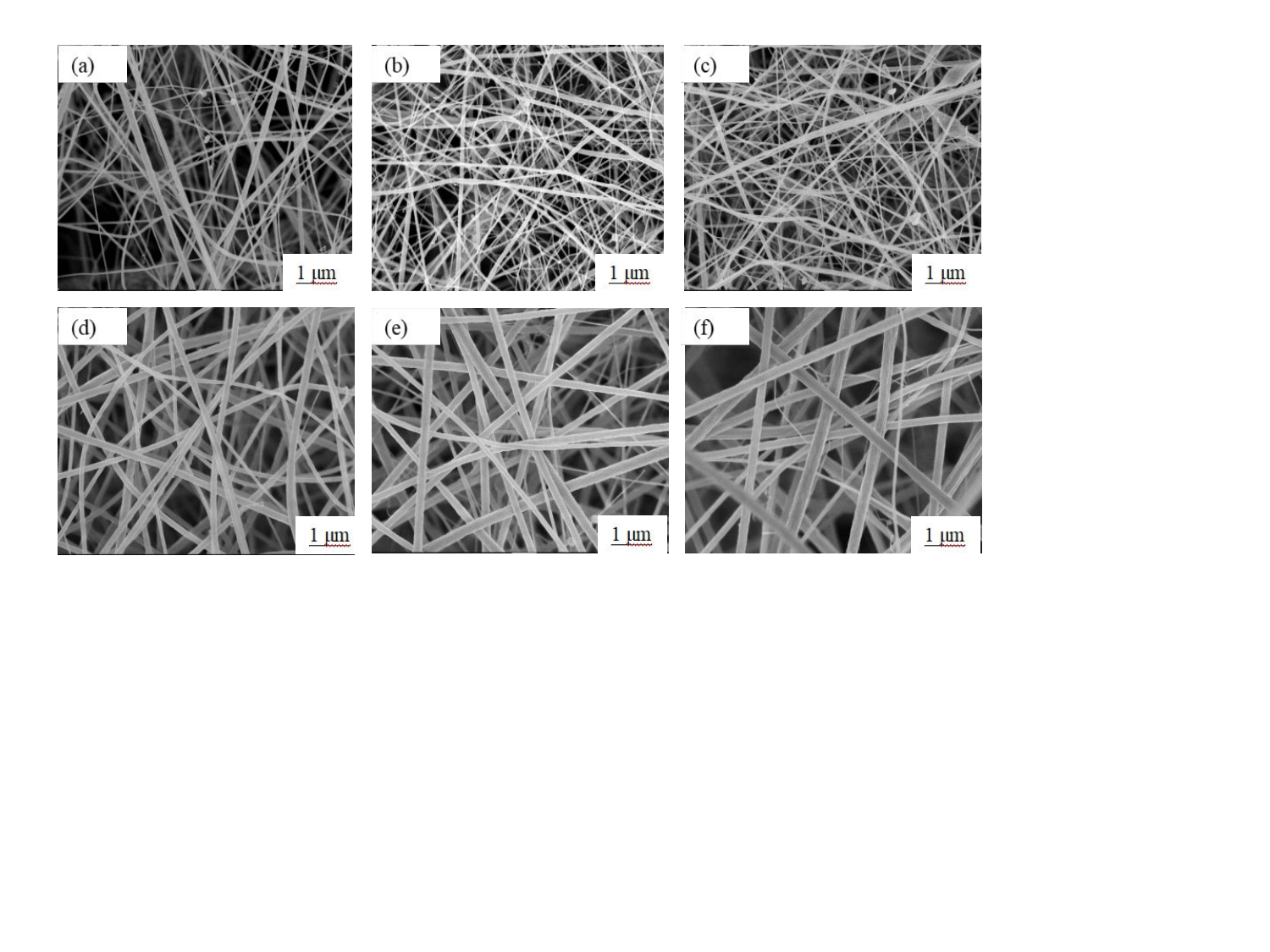

## Slide 4
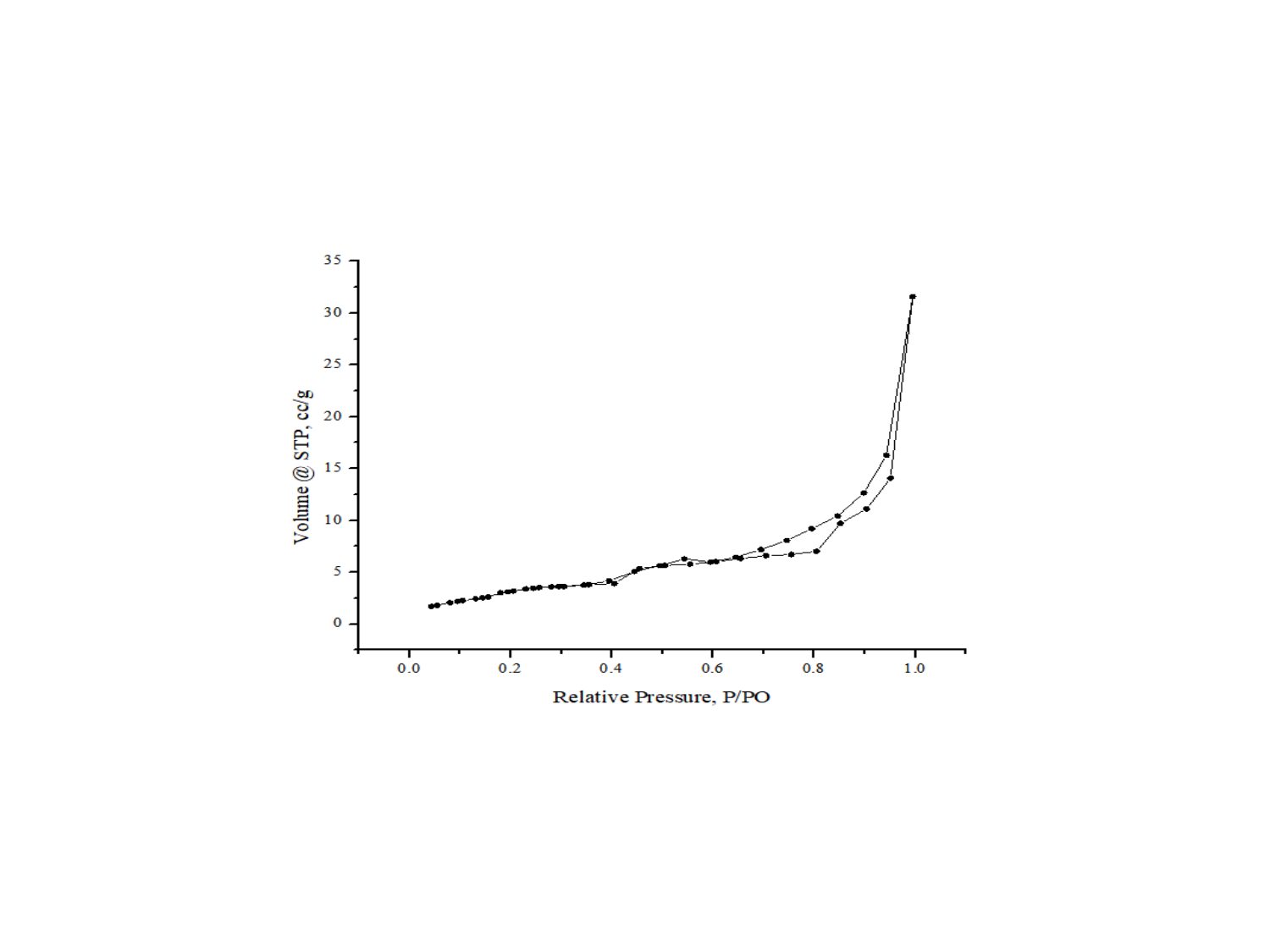

## Slide 5
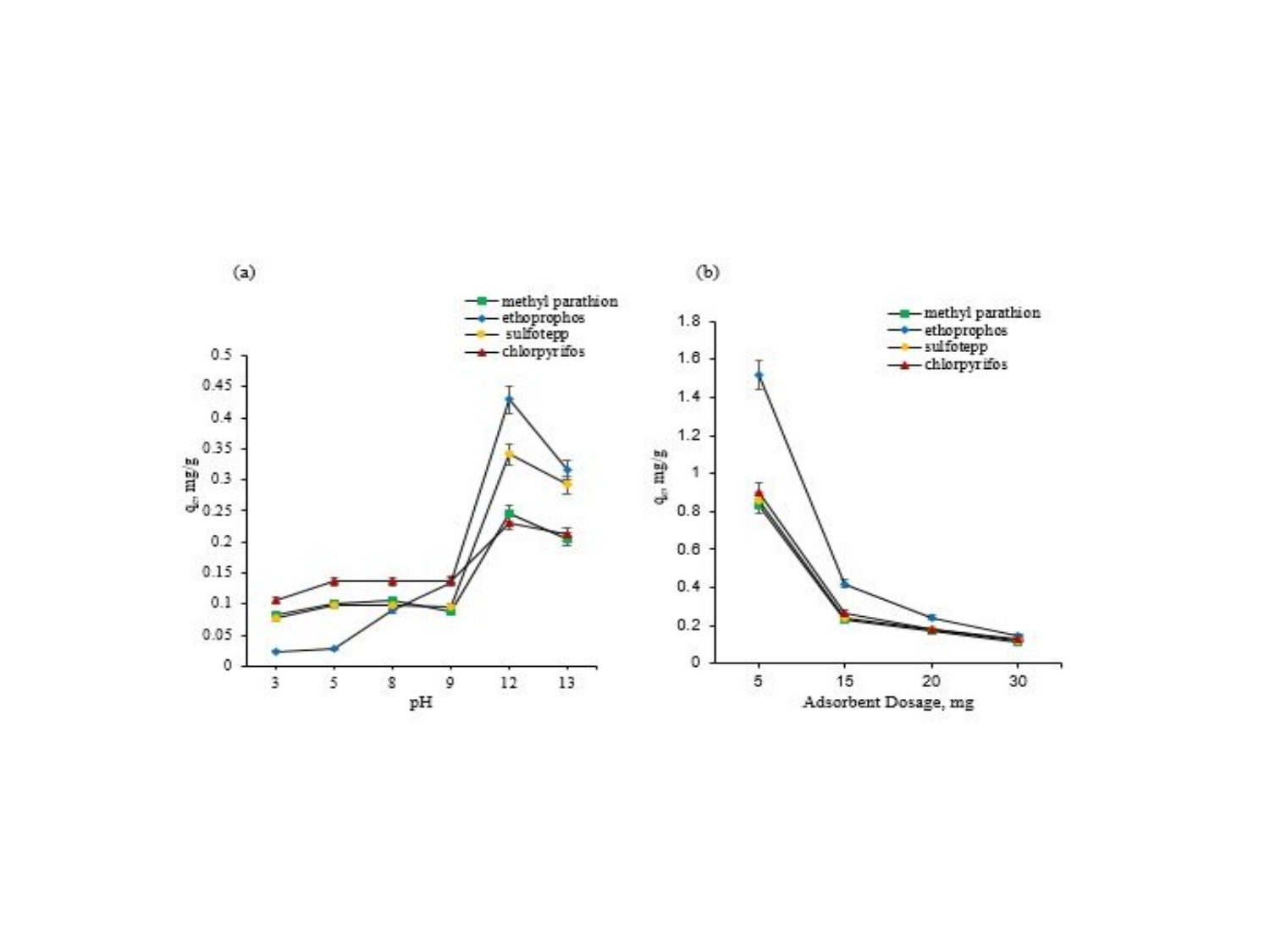

## Slide 6
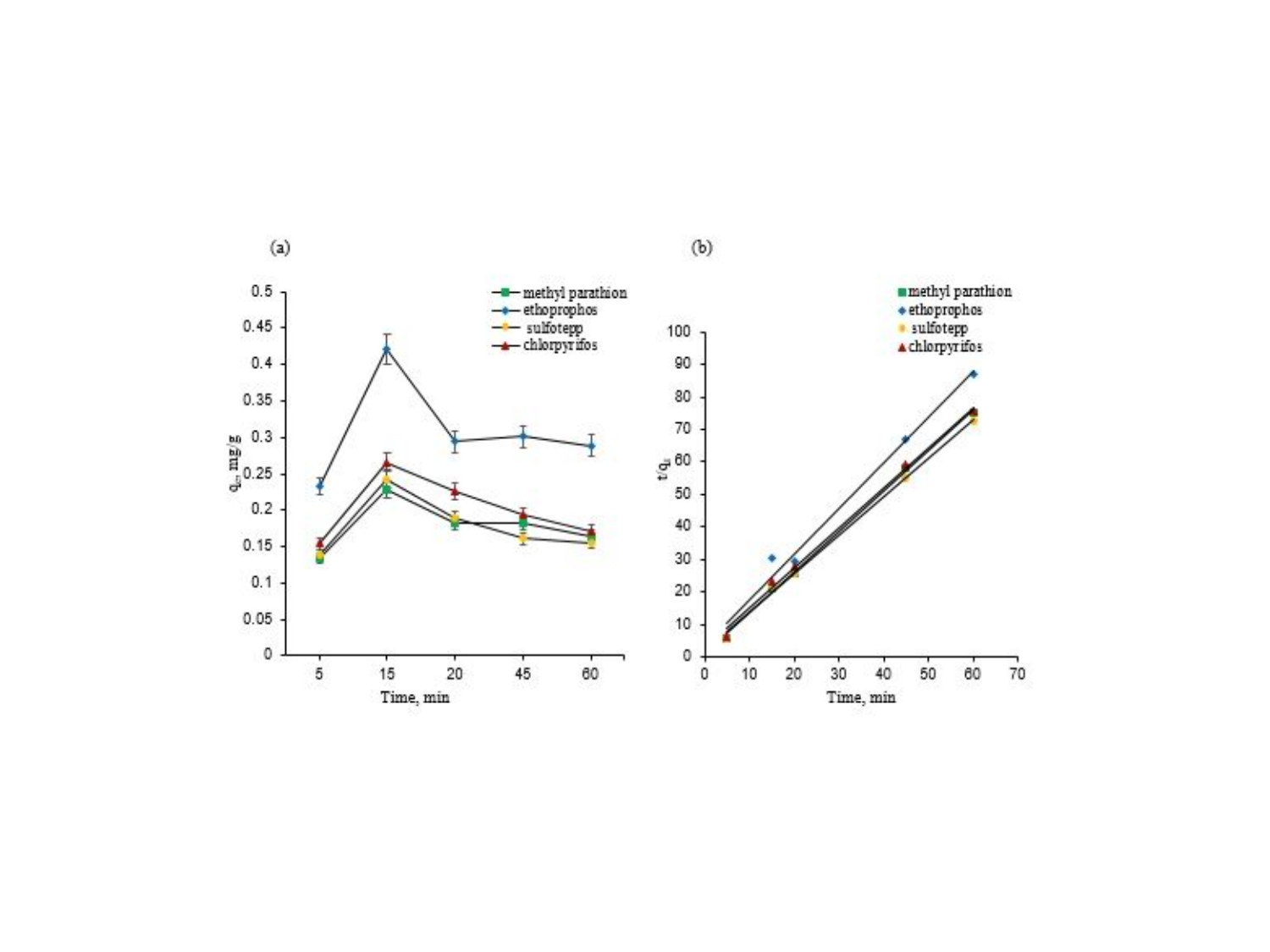

## Slide 7
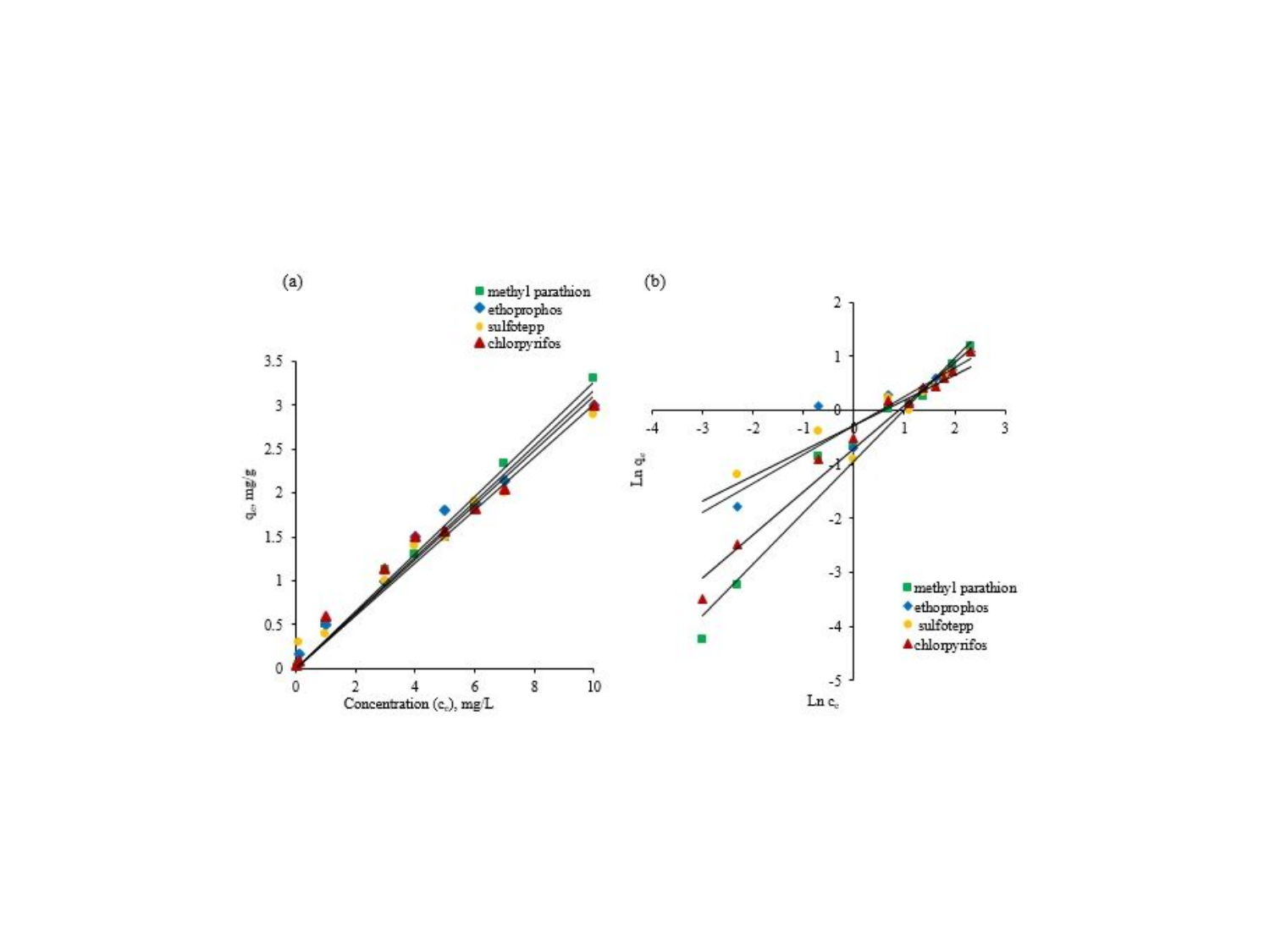

## Slide 8
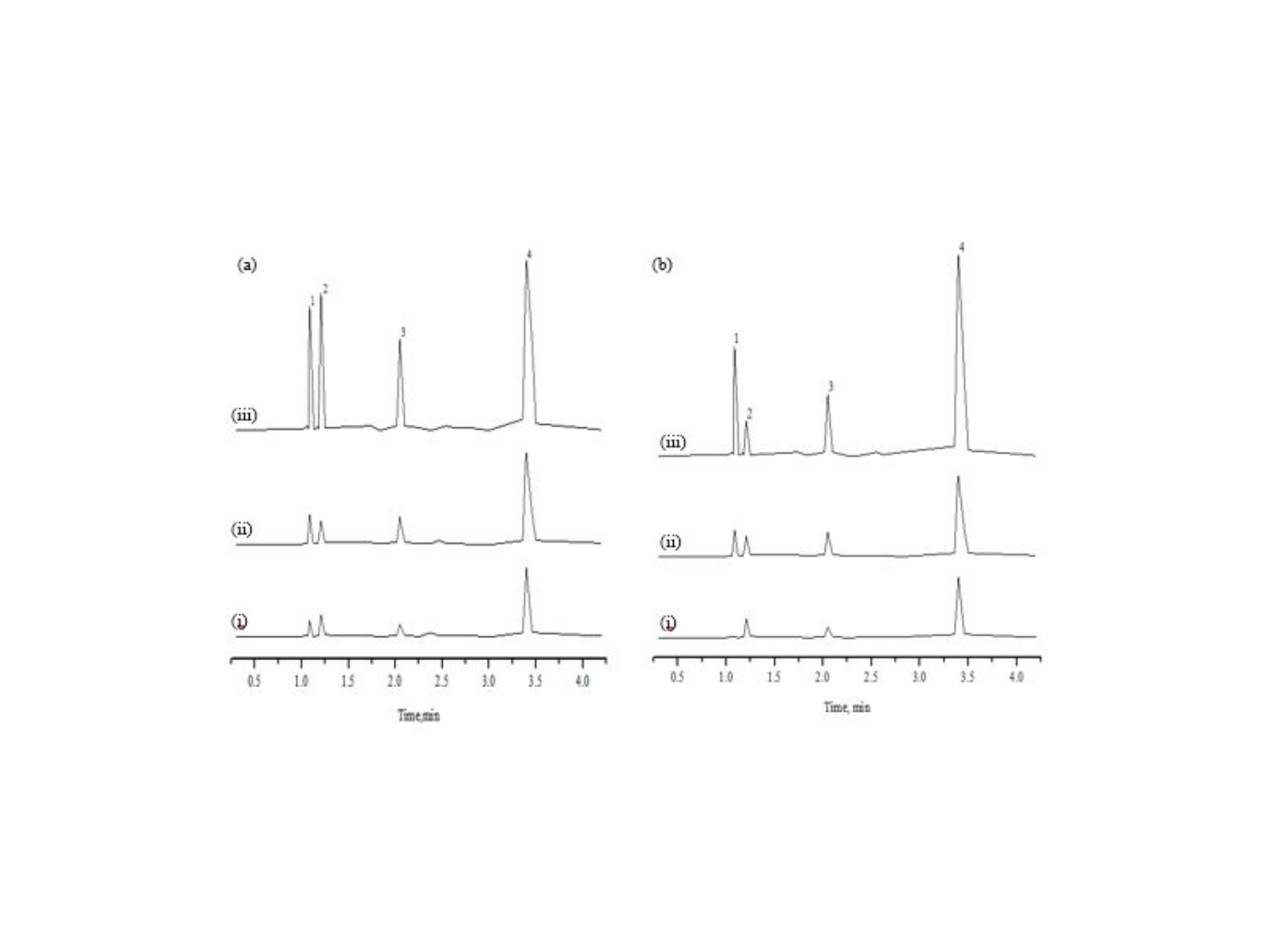

## Slide 9
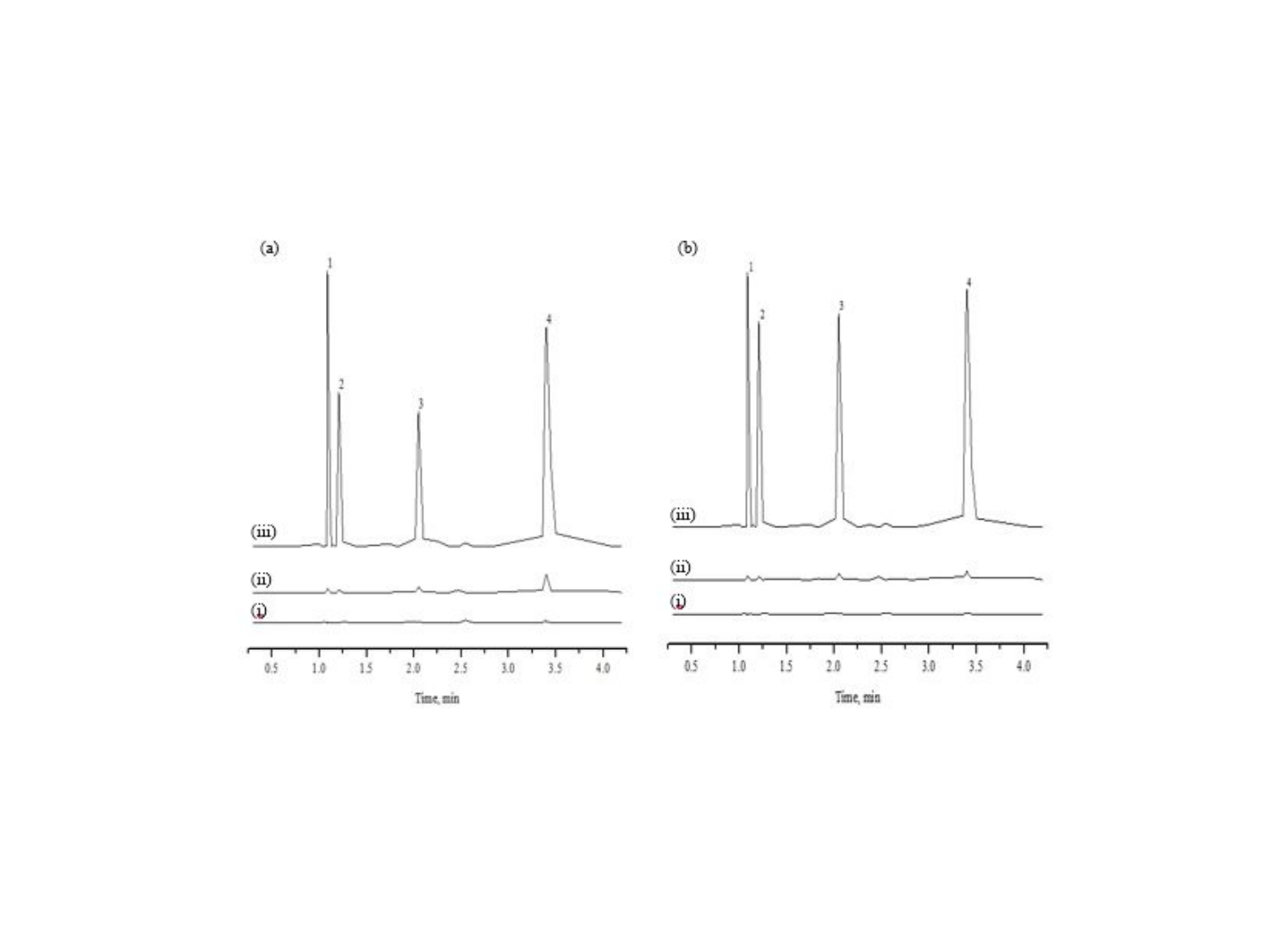

## Slide 10
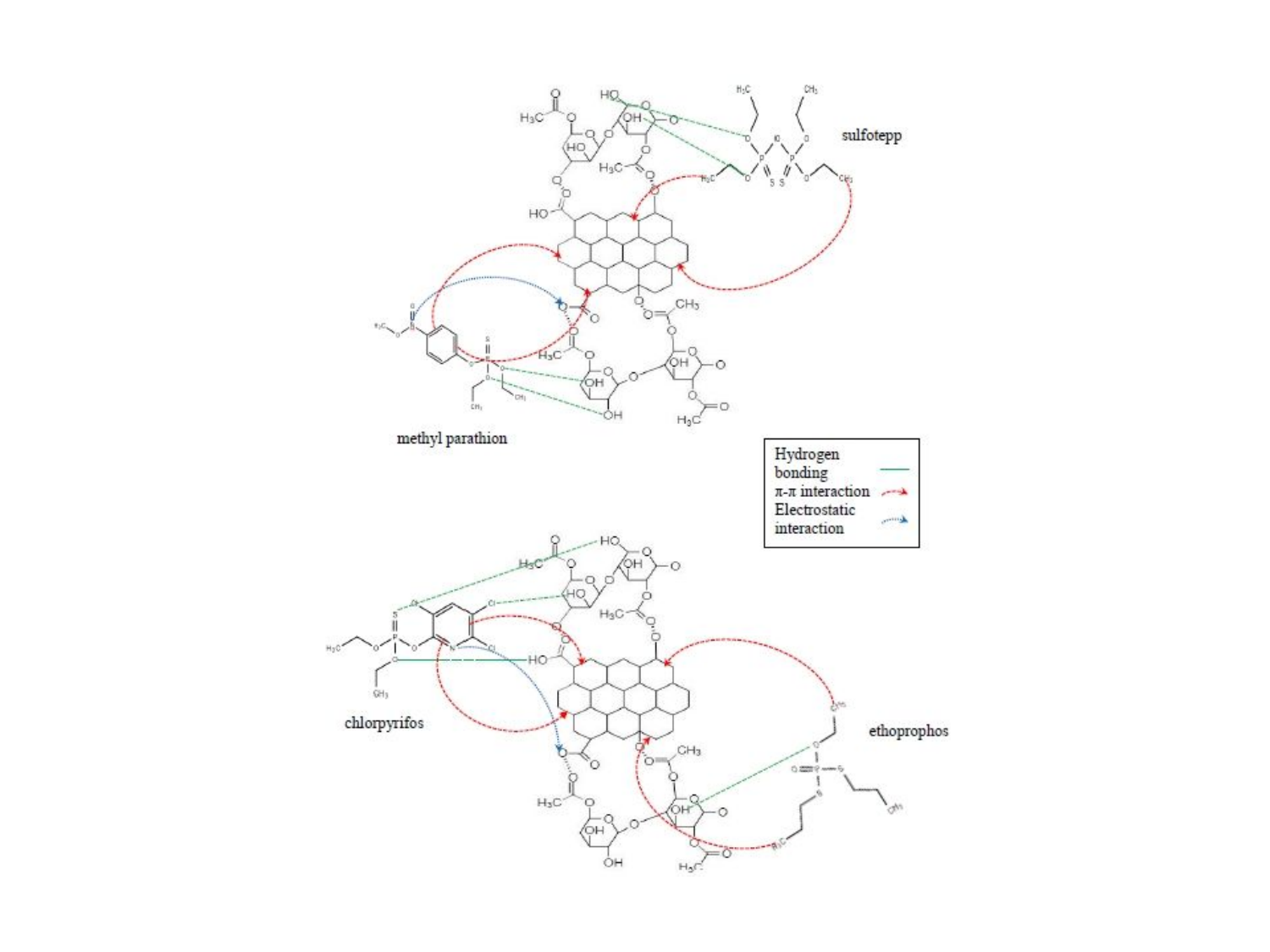

Supplement: Figures [file rsos192050supp2.pptx]
