# Supplementary material for: Superhydrophilic graphene oxide/electrospun cellulose nanofibre for efficient adsorption of organophosphorus pesticides from environmental samples
Source: R Soc Open Sci. 2020 Mar 11;7(3):192050. doi: 10.1098/rsos.192050 (PMC7137939; doi:10.1098/rsos.192050)
Supplement: FESEM raw data [file rsos192050supp3.pptx]

## Slide 1
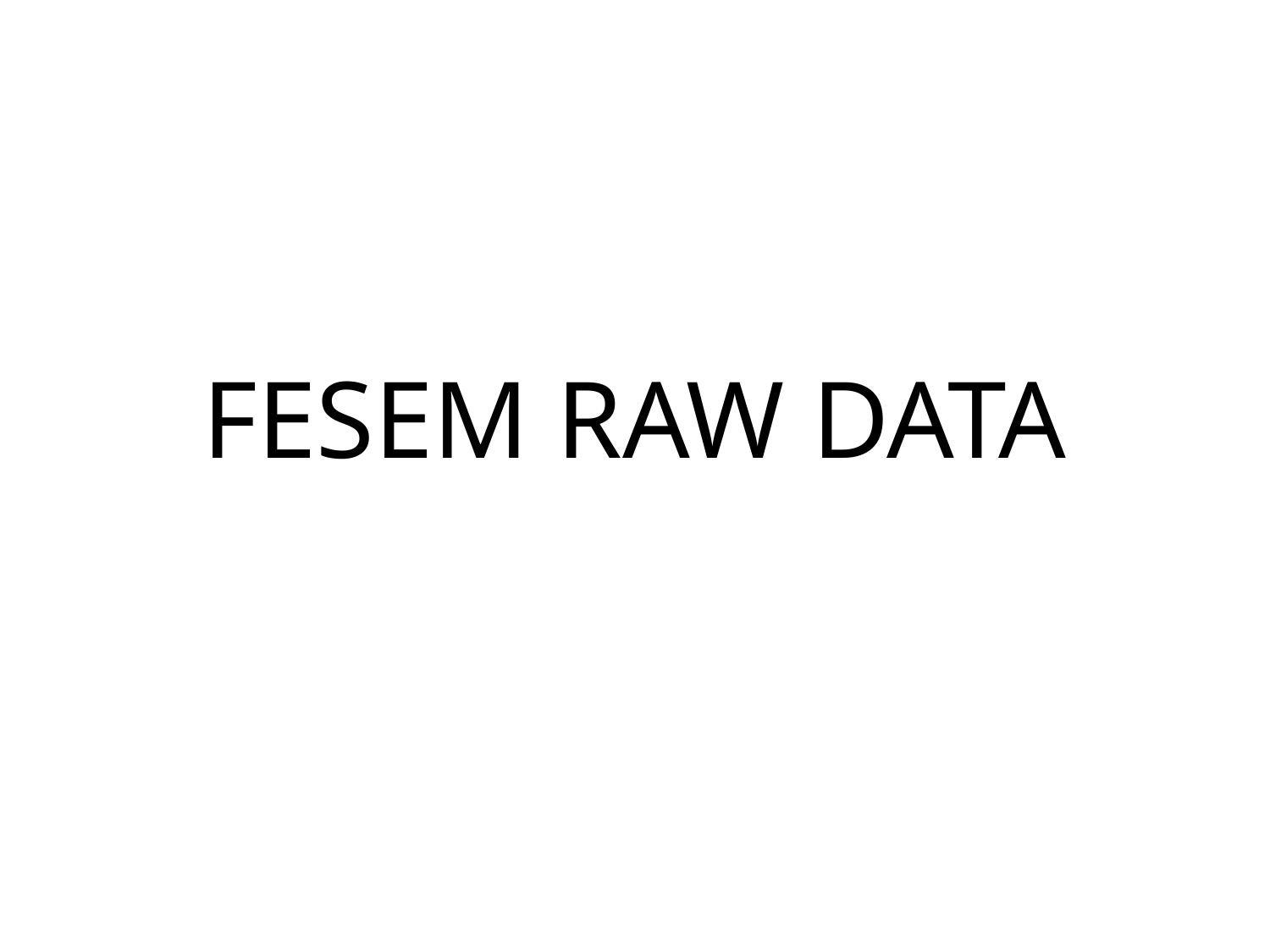

# FESEM RAW DATA

## Slide 2
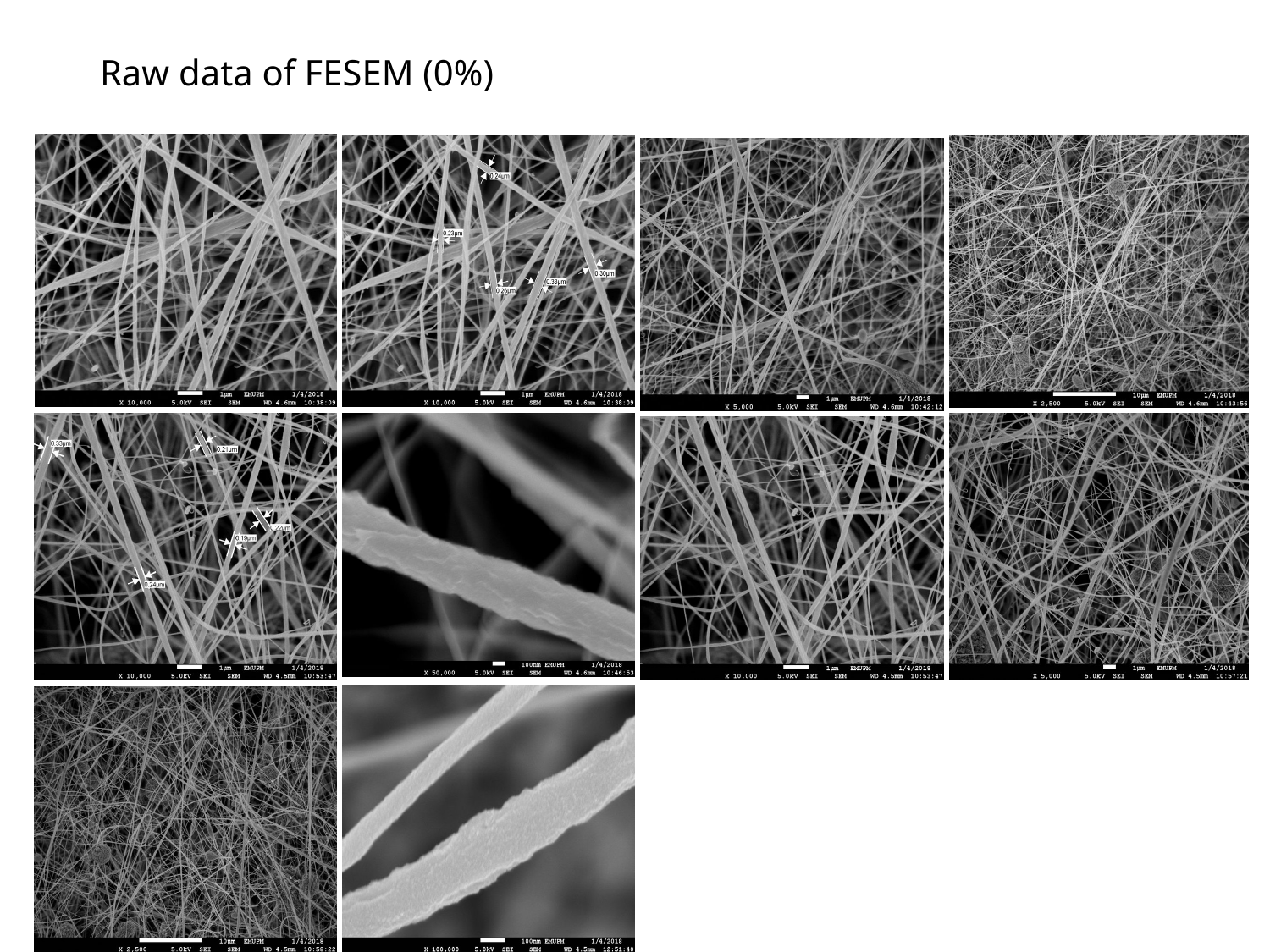

# Raw data of FESEM (0%)

## Slide 3
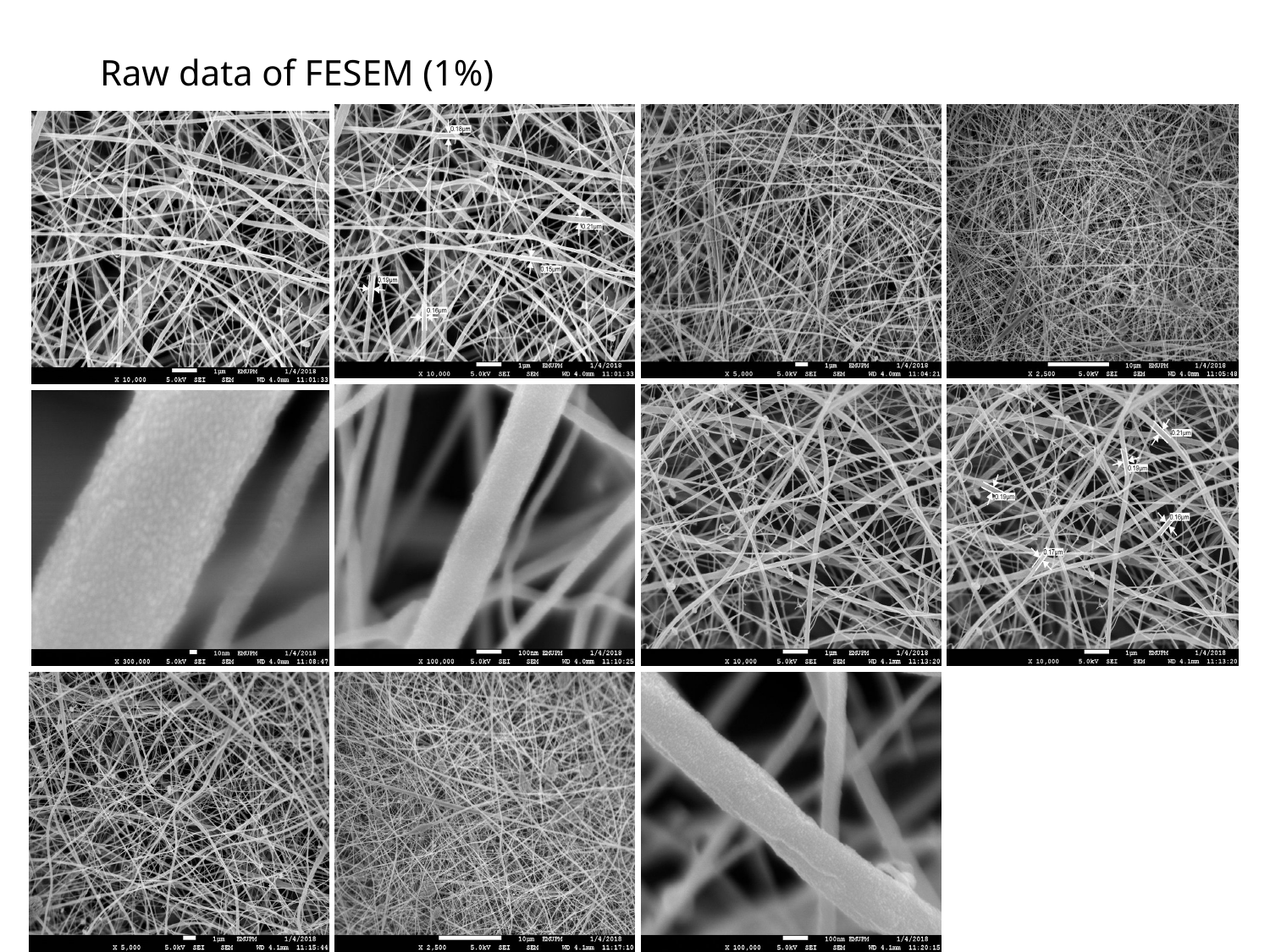

Raw data of FESEM (1%)

## Slide 4
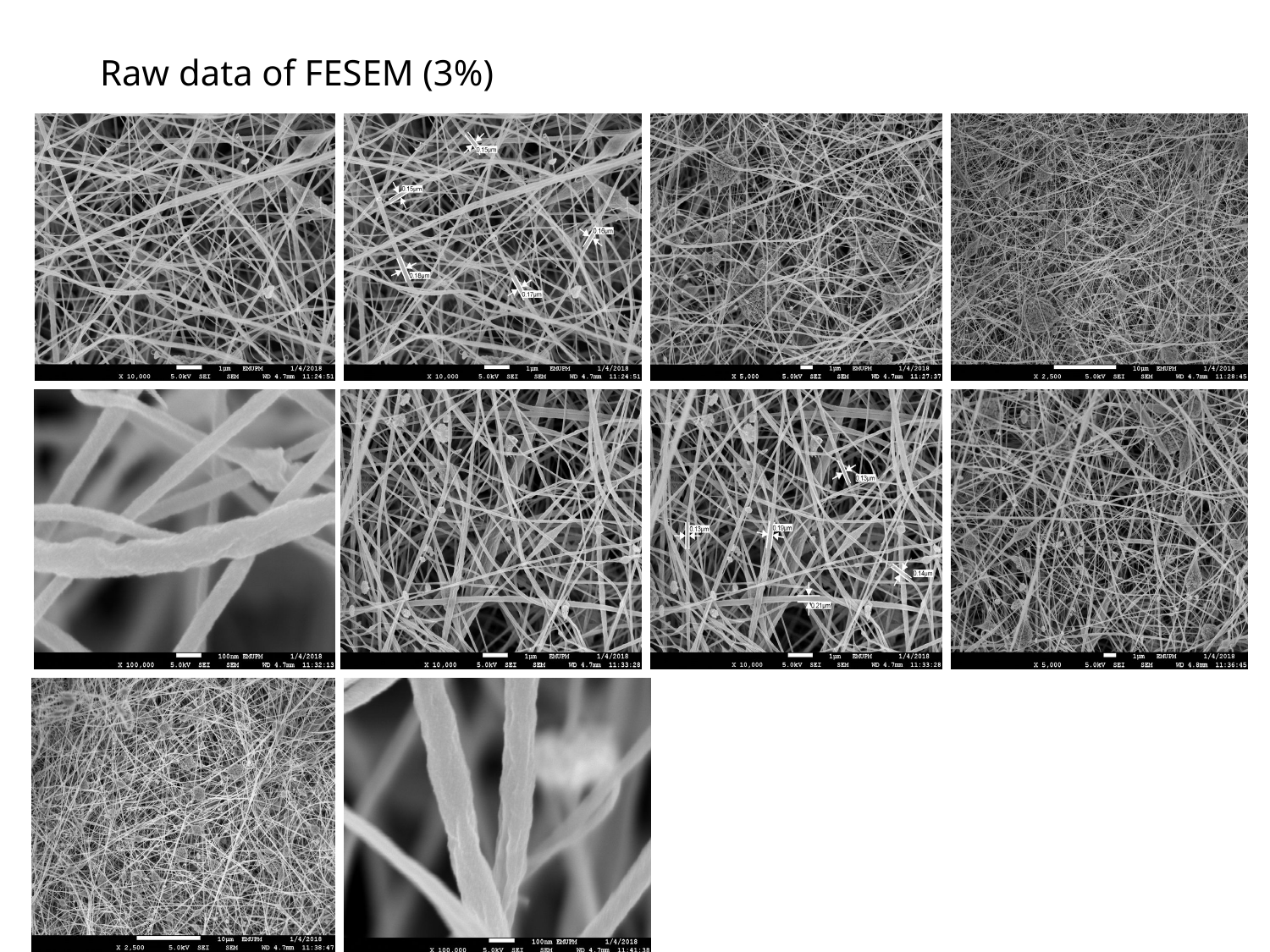

Raw data of FESEM (3%)

## Slide 5
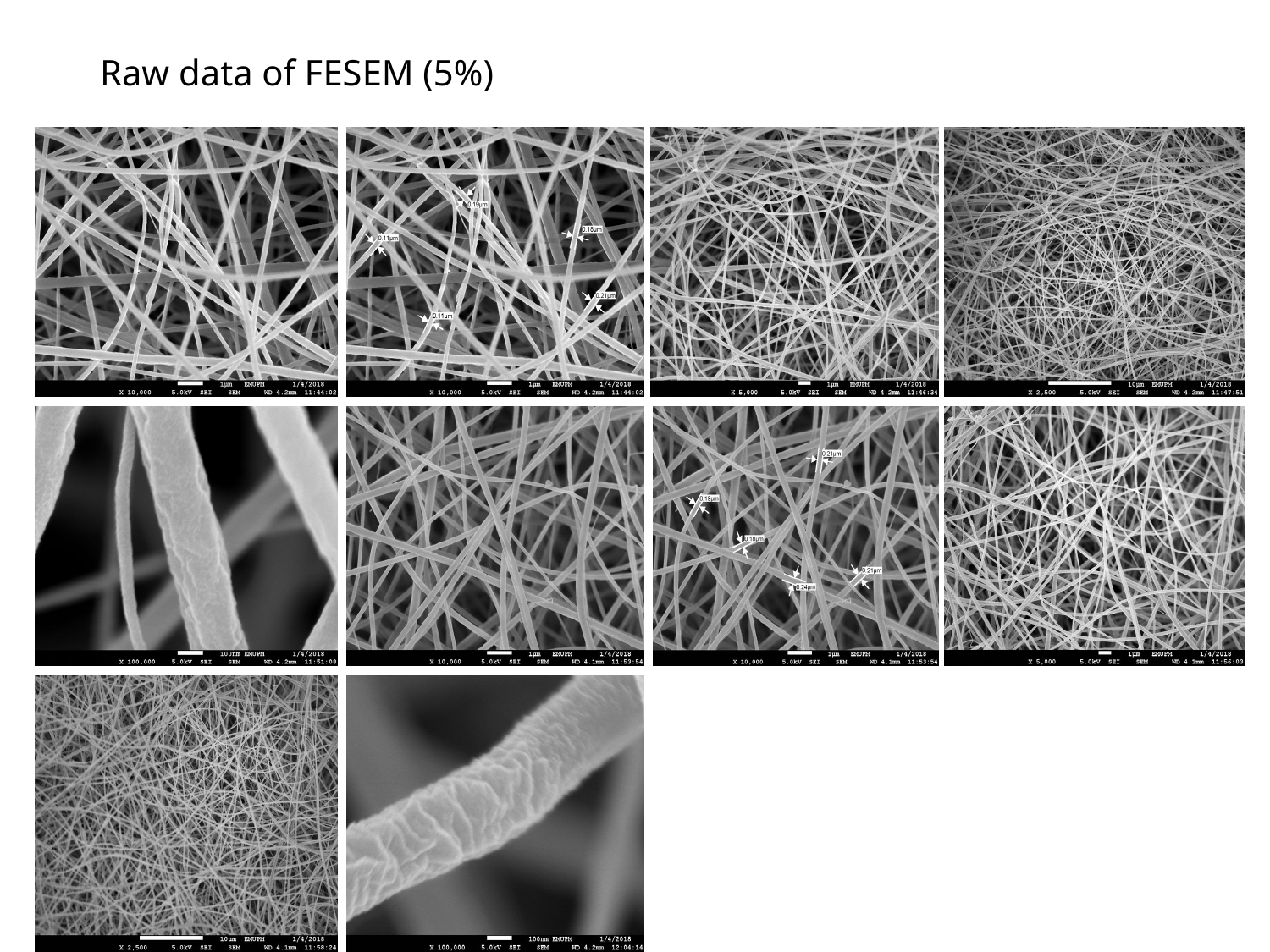

Raw data of FESEM (5%)

## Slide 6
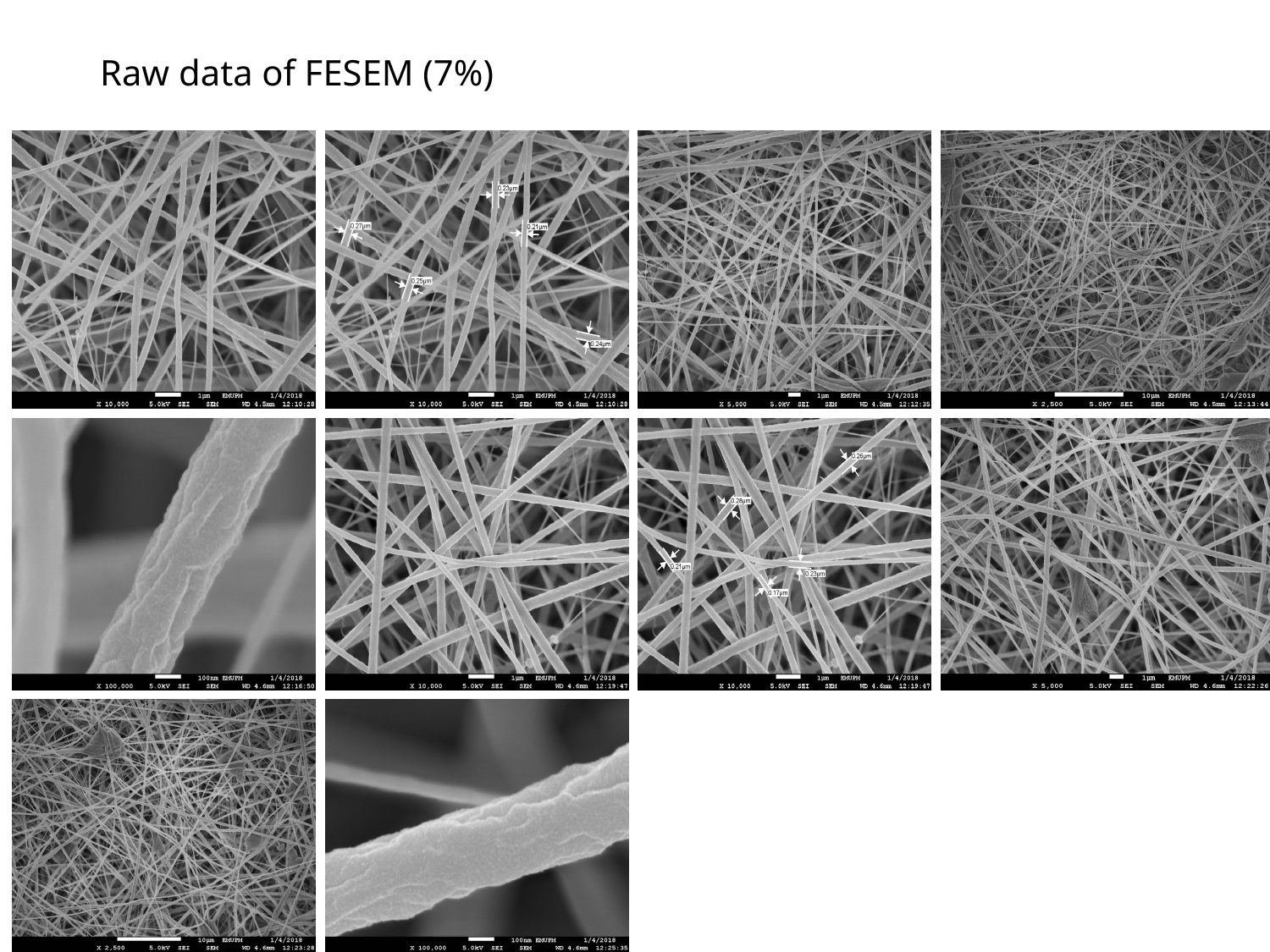

Raw data of FESEM (7%)

## Slide 7
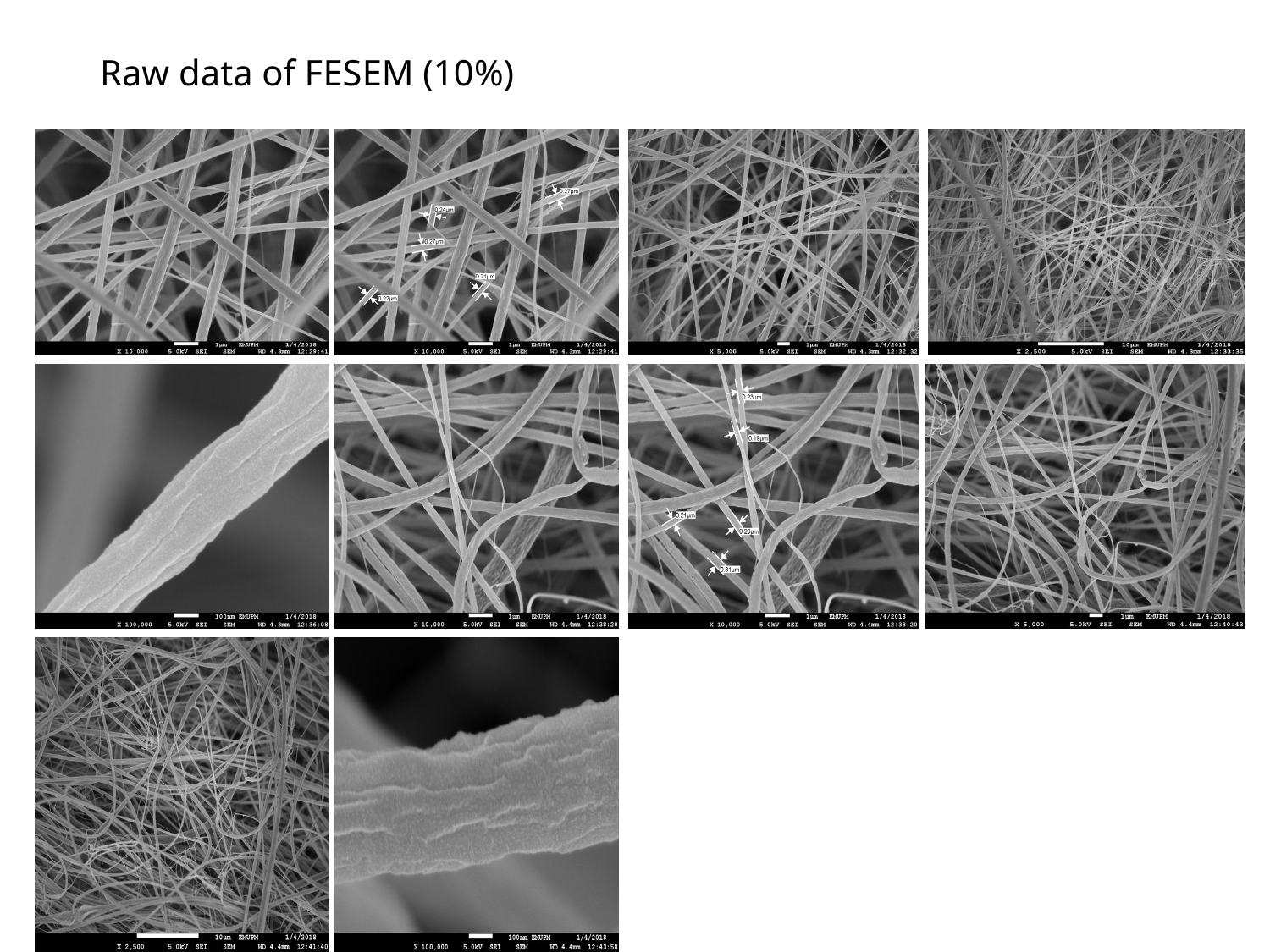

Raw data of FESEM (10%)
